
JOURNAL INFORMATION
==============================
NLM Title Abbreviation: ScientificWorldJournal
Journal ID: ScientificWorldJournal
NLM Journal ID: 101131163
Journal ID: TSWJ

The Scientific World Journal

ISSN: 2356-6140
EISSN: 1537-744X
Publisher: Wiley

ARTICLE INFORMATION
==============================
PMCID: PMC5956446
PMID: 15349566
DOI: 10.1100/tsw.2004.44
Article ID: 976849
Article version: 1
Subjects: Original Report

Uretero-Infundibuloplasty for Giant Hydronephrosis

Cain Mark P.
Vanderslice Richard
Gibbons M. Dave
Riley Hospital for Children, Department of Urology, Indiana University Medical Center, Division of Urology, Georgetown Medical Center, Washington, DC, USA
Academic Editor: Anthony Atala

Publication date: 2004
Electronic publication date: 2004 Jun 28
Volume: 4
First page: 438
Last page: 442
Copyright: Copyright © 2004 Mark P. Cain et al.
Copyright year: 2004
License: This is an open access article distributed under the Creative Commons Attribution License, which permits unrestricted use, distribution, and reproduction in any medium, provided the original work is properly cited.
License URL: https://creativecommons.org/licenses/by/3.0/

==============================
Original Report
*TheScientificWorldJOURNAL* (2004) 4 (S1), 438–442
ISSN 1537-744X; DOI 10.1100/tsw.2004.44

### Uretero-Infundibuloplasty for Giant Hydronephrosis

Mark P. Cain, M.D., Richard R. Vanderslice, M.D.,
and M. Dave Gibbons, M.D.

*Riley Hospital for Children, Department of Urology, Indiana University Medical Center;*
*Division of Urology, Georgetown Medical Center, Washington D.C.*

*Previously published in the Digital Urology Journal*

**A large cystic abdominal mass was identified on a prenatal ultrasound in a 37 gestational week female fetus. Antenatal fluid aspiration from the cyst was consistent with fetal urine. The cyst fluid rapidly reaccumulated, prompting induction of labor. Following vaginal delivery, radiographic evaluation revealed giant hydronephrosis with ureteropelvic junction (UPJ) obstruction. This anomaly was associated with a unusual variation of intrarenal anatomy, which prevented classic dismembered pyeloplasty. We report a case of in-utero detected giant UPJ obstruction and the unique reconstruction utilizing the concept of dismembered ureteroinfundibuloplasty.**

**DOMAIN**: urology

### CASE REPORT

A 36 year-old female had an abnormal antenatal sonogram in the 37th week of pregnancy revealing a female fetus with a large cystic abdominal mass (9.8x8x6 cm). Both kidneys were identified lateral to the mass, and neither collecting system was dilated. In-utero aspiration and drainage of the cystic mass yielded clear fluid with electrolytes consistent with fetal urine (Na 35 meq/L, C1 36 meq/L, 96 mOsm/kg). Amniotic fluid volume was normal and amniocentesis revealed 46 XX kariotype without abnormalities.

Repeat sonogram three days later demonstrated reaccumulation of the fluid within the cystic abdominal mass. Because of the persistent abdominal mass, labor was induced at 38 weeks gestation. Following uncomplicated vaginal delivery, physical exam revealed a smooth but firm, mid-abdominal mass. Genitalia were normal female. Postnatal abdominal ultrasound demonstrated that the right kidney was displaced posteriorly and malrotated. There was mild caliectasis in the right kidney, and no hydronephrosis on the left. A voiding cystourethrogram revealed anterior displacement of the bladder without evidence of vesicoureteral reflux. MRI scan with gadolinium (Figure 1) showed a large mid-abdominal ovoid cystic mass compressing the bladder, posterior and superior displacement of the left kidney, and posterior displacement of the right kidney. There were no renal parenchymal abnormalities or significant hydronephrosis. Delayed images demonstrated a communication between the cystic mass and the urinary tract.

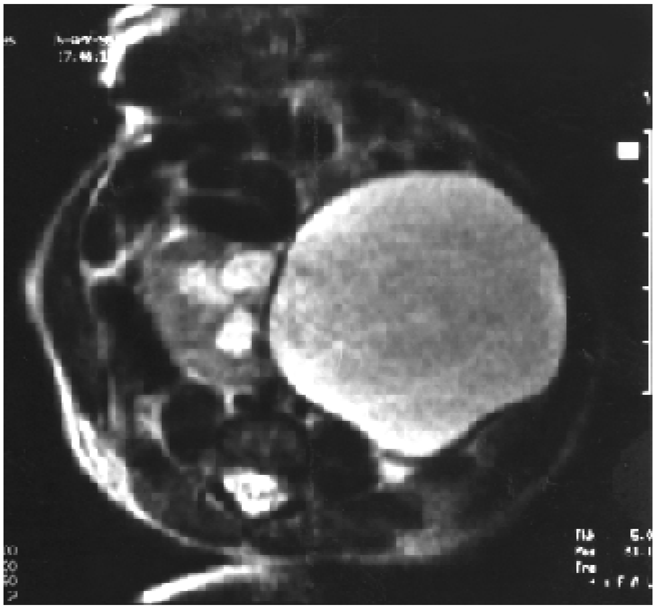

**FIGURE 1.** Preoperative MRI (T2 weighted image) demonstrating the disparity between the renal pelvic dilation and minimal calyceal dilation.

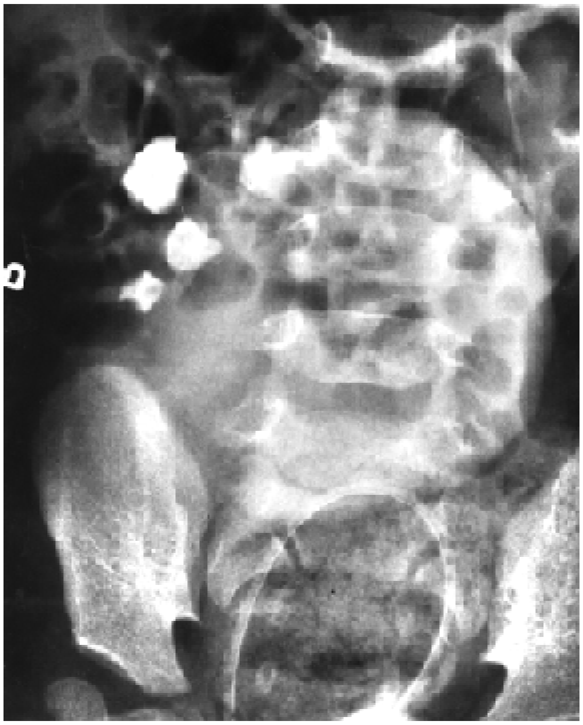

**FIGURE 2**. Preoperative intravenous urogram demonstrated the mildly dilated right calyces adjacent to the large cystic abdominal mass, which slowly filled with contrast. Note the absence of discernible communication between the calyces and pelvis.

An intravenous urogram (Figure 2) confirmed malrotation of the right kidney with accumulation of contrast in the adjacent cystic mass, which appeared to be retroperineal in location. Prior to surgical exploration, a right retrograde ureterogram verified the presence of the right ureter, and demonstrated continuity between the ureter and cystic mass (consistent with giant UPJ). Right flank exporation revealed a malrotated, non-hydronephrotic kidney and massively distended right renal pelvis anterior to the kidney. The dilated pelvis communicated with the intrarenal collecting system via four markedly attenuated infundibulae (Figure 3). The ureter entered the posterior mid-portion of the renal pelvis. The entire renal pelvis was excised, leaving the proximal ureter and two cuffs of renal pelvis, each subtending two infundibulae. Infundibuloplasty was conducted by joining the superior and inferior cuffs and ureteroinfundibuloplasty was then accomplished by anastomosing the ureter to the dependent portion of the lower infundibulum with 6-0 PDS.

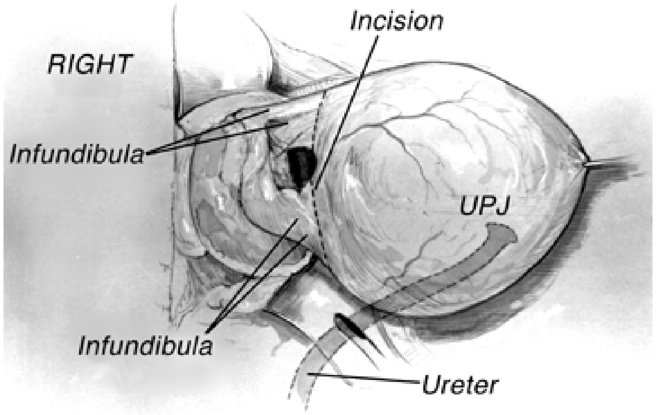

**FIGURE 3**. Drawing of the intraoperative findings. Well preserved renal parenchyma was attached to the massively dilated pelvis via four attenuated infundibulae. Ureter is coursing posterior to the pelvis with a high insertion. The line of resection between the pelvis and the infundibulae is shown. *Illustration by P. Stone*

Following a three week period of urinary diversion with a ureteral stent and nephrostomy tube, an intravenous pyelogram demonstrated prompt renal function and a nondilated neopelvis and ureter (Figure 4).

### DISCUSSION

Giant hydronephrosis is a rarely encountered entity. Approximately 200 cases have been reported, but none have been identified antenatally. Giant hydronephrosis is defined when the adult renal pelvis contains more than one liter of urine [1]or, in childhood, more than one day of urine volume [2]. Pfister et al [3] use the following radiographic criteria: the kidney occupies a hemiabdomen, meets or crosses the midline and is at least 5 vertebral bodies in length.

The most common cause of giant hydronephrosis is a high insertion of the ureter resulting in ureteopelvic junction obstruction. Approximately two thirds of these kidneys have preserved renal function and are amenable to repair [3]. This unexpectedly high salvage rate may be due to the "protective" effect of the compliant, overdistended pelvis on the renal parenchyma [4]. In this case, the diagnosis was unclear prior to exploration. A nonurologic retroperitoneal mass (mesenteric cyst, teratoma. etc.) was considered, with iatrogenic communication between the right kidney (MRI and IVP demonstrated communication between the right collecting system and the mass). The disparitybetween mi ld calyceal dilation and the marked size of the cystic mass mitigated against the diagnosis of hydronephrosis. The

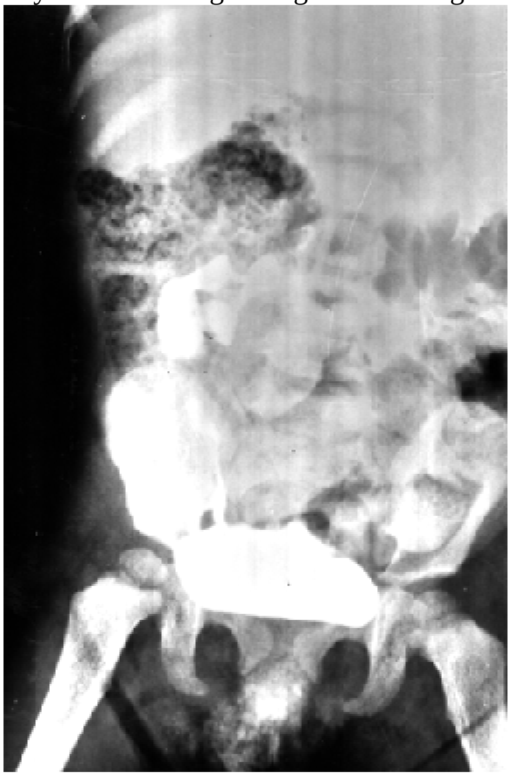

**FIGURE 4.** Postoperative intravenous urogram demonstrated prompt excretion of contrast into the distal collecting system.

unique anatomy of the collecting system (i.e. four attenuated infundibulae preventing complete transmission of renal pelvic pressure into the intrarenal collecting system) accounted for the minimal dilation of the calyces. The true renal pelvis was dramatically dilated due to a UPJ obstructrion secondary to a high insertion of the ureter. Reconstruction required complete resection of the renal pelvis and ureteroinfundibuloplasty to allow close approximation of the attenuated infundibulae.

Antenatal diagnosis was inaccurate in providing a precise etiology for the cystic abdominal mass. This variant of giant hydronephrosis had an unusual anatomic relationship between the intrarenal collecting system and renal pelvis, obscuring the diagnosis and making the reconstruction challenging. A previously undescribed procedure, ureteroinfundibuloplasty, was required to create a neopelvis. Ureteroinfundibuloplasty provided a method of i) reconstruction of the neopelvis, ii) avoiding reconstructive problems associated with the overdistended native renal pelvis, and iii) providing dependent ureteral drainage. This technique should be considered and applied in variations of ureteropelvic obstruction that are associated with giant hydronephrosis.

### REFERENCES

1. Earlham MS: Giant Hydronephrosis. J Urol 63: 195, 1950.
2. Yang W., Shen S., Wu C.: Hydronephrosis and Giant Hydronephrosis. Chin Med J 77: 257-259, 1958.
3. Crooks K., Hendren H., Pfister R.: Giant Hydronephrosis in Children. J Ped Surg 14(6): 844-850, 1979.

4. Koff S.: Pathophysiology of Ureteropelvic Junction Obstruction: Clinical and Experimental Observations. Urol Clin NA 17(2): 263-272, 1990.

---

**This article should be referenced as follows:**

Cain, M.P., Vanderslice, R.R., and Gibbons, M.D. (2004) Uretero-infundibuloplasty for giant hydronephrosis. *TheScientificWorldJOURNAL* **4 (S1)**, 438–442.

**Handling Editor:**

Anthony Atala, Principle Editor for *Urology* — a domain of *TheScientificWorldJOURNAL*.

---
